# Lessons Learned: Gastric Motility Assessment During Driving Simulation

**DOI:** 10.3390/s19143175

**Published:** 2019-07-19

**Authors:** Nenad B. Popović, Nadica Miljković, Kristina Stojmenova, Grega Jakus, Milana Prodanov, Jaka Sodnik

**Affiliations:** 1 School of Electrical Engineering, University of Belgrade, Belgrade 11000, Serbia; 2 Faculty of Electrical Engineering, University of Ljubljana, Ljubljana 1000, Slovenia

**Keywords:** electrogastrography, driving simulation, simulator sickness, open-source hardware

## Abstract

In the era of technological advances and innovations in transportation technologies, application of driving simulators for the investigation and assessment of the driving process provides a safe and suitable testing environment. Although driving simulators are crucial for further improvements in transportation, it is important to resolve one of their main disadvantages–simulator sickness. Therefore, suitable methods for the assessment of simulator sickness are required. The main aim of this paper was to present a non-invasive method for assessing simulator sickness by recording gastric myoelectrical activity–electrogastrography. Open-source hardware for electrogastrography together with recordings obtained in 13 healthy volunteers is presented, and the main aspects of signal processing for artifact cancellation and feature extraction were discussed. Based on the obtained results, it was concluded that slow-wave electrical gastric activity can be recorded during driving simulation by following adequate recommendations and that proposed features could be beneficial in describing non-ordinary electrogastrography signals.

## 1. Introduction

Driving simulation is a tool that provides an alternative to on-road testing in a safe, reliable, and efficient manner [1]. Since its first use during World War II [2], it has been widely used for training and assessment of driving skills. This method, compared to traditional field testing, offers many advantages, such as safety, simplicity, cost efficiency, and variability regarding driving conditions [2]. However, one of its pitfalls is simulator sickness (SS), a condition including a variety of unpleasant symptoms that influences quality of experience, increases rate of drop-outs, and limits the effectiveness and duration of the training [3,4]. It can be described by the following physical sensations: headache, sweating, dry mouth, drowsiness, disorientation, vertigo, nausea, dizziness, vomiting, etc. [4,5]. Therefore, SS needs to be addressed and minimized in order to enable future advances in the field of simulator design. In order to fully understand and reduce SS problems, appropriate assessment is required. Simulator sickness is commonly assessed with self-reporting. There are a number of available questionnaires for rating the level of sickness by evaluating different physical sensations. The most commonly used methods are the simulator sickness questionnaire (SSQ) and Fast Motion Sickness Scale (FMS), which can be used for the quantification of subjective simulator sickness experience, and oculomotor discomfort, nausea and disorientation as its subcategories [6,7]. However, these methods do not provide information on physiological parameters, and the downside of its application is that it is not sensitive enough to discriminate between simulators [7]. In the review article [8], it is stated that previously used physiological measurements include: (1) electroencephalography (EEG); (2) electrocardiography (ECG); (3) temperature; and (4) galvanic skin response (GSR). All these physiological measurements proved sensitivity to SS. Namely, SS occurrence was followed by: (1) increased activity in δ and decreased activity in α, β, and θ waves (EEG studies); (2) increased heart rate (ECG studies); (3) decreased skin temperature; and (4) increased skin conductance (GSR studies) [8]. Although all these physiological methods proved its efficacy, they are not able to identify SS occurrence and severity, and none of them provided direct assessment of the most commonly experienced SS symptom-nausea. In order to assess nausea directly, we propose non-invasive gastric motility monitoring using electrogastrography (EGG).

Non-invasive recording of myoelectrical potentials from stomach smooth muscles (EGG) was first described in the literature by Alvarez in 1921 [9]. Since then, the major progress in that area has been made during the past three decades, but the application of EGG in clinical practice is still limited [10,11]. The main component of EGG signals consists of a slow-wave, i.e. sinusoidal signal with a frequency ranging from 2 cpm (cycles-per-minute) to 4 cpm in healthy subjects-normogastric range [12,13]. In previous studies [14,15,16], a shift in the frequency of slow-wave activity from normogastric range was reported in healthy subjects as a consequence of nausea occurrence. This phenomenon is also called gastric dysrhythmia [17]. The same results were obtained during the assessment of nausea as a result of motion and simulator sickness [14,15,16,18,19,20]. To the best of our knowledge, all published EGG studies for SS assessment were performed under static conditions (in the absence of movement artifacts). Our aim was to explore EGG usability in a dynamic environment by the application of a haptic simulator platform. We were motivated to test in a driving simulator with haptic feedback, since its employment can decrease probability of SS occurrence [20]. One of the main challenges for assessment in a driving simulator with a haptic platform is the vulnerability of an EGG signal to motion artifacts [12]. This sensitivity is a consequence of EGG nature, which is characterized by low amplitude (200–500 µV) compared to other electrophysiological signals, and by the frequency spectrum with values close to 0 Hz (0.016–0.15 Hz) [21]. With high gain values (> 1000) there is a concern that interference with other physiological signals like electromyography(EMG), ECG, breathing, and body movements could be increased resulting in noise that completely covers the relevant EGG information [12]. Consequently, filtering is essential in order to preserve the signal of interest for successful EGG measurements.

In this paper, we present open-source hardware dedicated for the acquisition of EGG signals. We also report on its performance in SS assessment when driving in Nervtech’s 4DOF motion-based car driving simulator with haptic feedback (Nervtechd.o.o, Trzin, Slovenia) [22]. 

The three main research questions are:

- Is it possible to reliably acquire slow-wave activity using a custom-made EGG sensing system during driving simulation?
- What EGG parameters are suitable for the analysis of recorded signals and, is there a correlation between them and subjective sickness assessment?
- Is there a clear difference in signals for resting, and the motion and no-motion drive?

## 2. Methods

Our protocol for SS assessment was tested in 13 healthy volunteers and included three testing conditions: (1) resting; (2) drive with haptic feedback (motion drive); and (3) drive without haptic feedback (no-motion drive). EGG signal analysis consisted of two parts. The first part included pre-processing methods for artifact cancellation, and the second part incorporated methods for the extraction of the following features: (1) dominant frequency (DF); (2) median frequency (MF); (3) crest factor (CF); (4) root mean square (RMS); and (5) percentage of spectral power in the normogastric range (2–4 cpm). All parameters were calculated for three testing conditions, and the results were thoroughly compared and discussed. In addition to EGG-based measurements, the subjects filled out Simulator Sickness Questionnaires (SSQ)—a widely used subjective measure to quantify simulator sickness [7]. Qualitative measures were compared to EGG-based quantitative parameters. On the basis of the tests performed and the results obtained, a detailed discussion on lessons learned is provided, with recommendations for the future application of EGG for SS assessment in driving simulators.

### 2.1. Participants

This study was conducted in Ljubljana, Slovenia, in compliance with the Code of Ethics of the University of Ljubljana, which provides guidelines for studies involving human beings and is in accordance with the Declaration of Helsinki.

The subjects were recruited from generally healthy Slovenian citizens, mostly from the students and staff of the University of Ljubljana. The exclusion criteria were pregnancy, disorders of gastrointestinal tract and vestibular system, so as other chronic and acute pathologies. All subjects reported that they did not use any medications at least one week prior to testing [18]. 

EGG signals were recorded in healthy subjects during driving simulation using the presented open-source EGG sensing system. Prior to the recording, the researchers explained the recording protocol to the subjects and presented the basic properties of the driving simulator. The test study group consisted of 13 healthy participants (ID1-ID9 and IDN1-IDN4), 4 females and 9 males, with age ranging from 23 years to 47 years (mean 29 ± 8 years), weight from 49 kg to 115 kg (mean 73 ± 19 kg), and height from 160 cm to 192 cm (mean 177 ± 8 cm). In Table 1., demographic data for all of the subjects included in the protocol is presented.

### 2.2. EGG Sensing System

In this chapter, the architecture of open-source hardware for the acquisition of EGG signals is presented. Low amplitude of EGG signals dictates that high gain should be obtained. Regardless of the fact that EGG could be recorded with a relatively low sampling frequency (< 4 Hz), low-pass (LP) filters are needed for the cancellation of high-frequency components coming from other physiological signals as well as possible artifacts originating from power hum. The most challenging step is baseline drift removal because it requires a high-pass (HP) filter with a cutoff frequency low enough to preserve the EGG frequency spectrum (close to 0 Hz, i.e. DC). Having that in mind, hardware was designed out of three main components: (1) amplification; (2) a HP filter; and (3) a LP filter. Due to the vulnerability of EGG recordings to artifacts, the application of more than one recording channel was indicated and therefore a three-channel EGG architecture was proposed [20]. Each channel had the same electrical components and configuration. The electrical circuit was realized on a breadboard, and DC power supplies as well as 16-bit A/D conversion were provided from the NI ELVIS II (National Instruments Inc., Austin, TX, USA) workstation. The sampling frequency was set to 2 Hz as recommended in [21].

**Figure 1:**
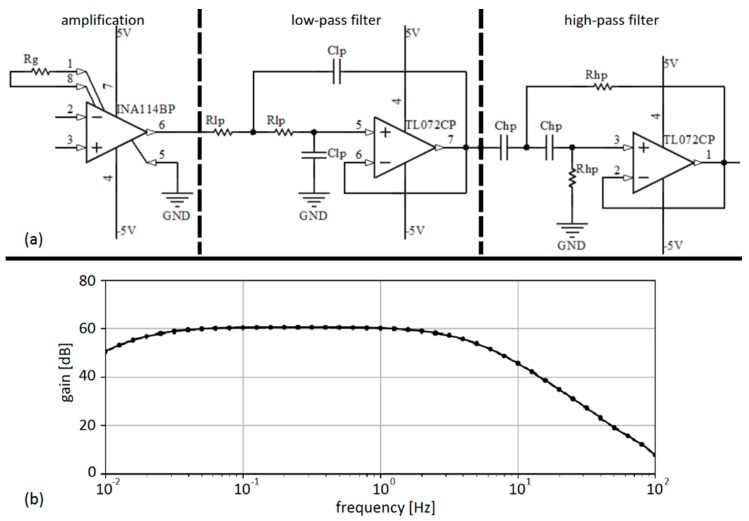
(**a**) Schematics for one channel of the EGG device. (**b**) Frequency characteristic for channel 1.

The schematic for one EGG channel is presented in Figure 1. (panel a). For amplification, an INA114 instrumentation amplifier was used in combination with an R_g_ resistor for gain setting–G = 1000 (specifications are given in Table 2). Both HP and LP filters were designed in Sallen–Key topology as active 2^nd^ order filters with cut-off frequencies 0.014 Hz and 4.820 Hz, respectively. For the implementation of the filters, a TL072CP dual operational amplifier was used.

Frequency characteristics in log-log scale presented on panel (b) in Figure 1 were obtained by an automated system for frequency response measurement based on free software tools [22]. The system consisted of a 33220A waveform generator (Keysight Technologies, Inc. Santa Rosa, CA, USA), a TBS 1052B-EDU digital oscilloscope (Tektronix, Inc. Beaverton, OR, USA), a personal computer equipped with the Ubuntu 16.04 LTS operating system, and an E3630A DC power supply (Keysight Technologies previous Agilent). The characteristics were recorded in 41 points ranging from 10 mHz to 100 Hz with a custom-made Python script (Python Software Foundation, Wilmington, DE, USA). The difference between channels was less than 2%, so only the characteristics for channel 1 are presented.

Our setup included a Force Sensing Resistor (FSR) as a pushbutton for manual synchronization between the driving simulator and EGG recordings. FSR was digitized by an ELVIS A/D connector (NI) together with three EGG channels.

### 2.3. Driving Simulator

We used Nervtech’s 4DOFmotion car driving simulator that includes a racing car seat and a three-pedal set with a steering wheel by Fanatec (Endor, Landshut, Germany). The display is composed of three curved 49-inch screens and SCANeR software (AVSimulation, Boulogne-Billancourt, France). This device is capable to deliver realistic simulation of different roads and driving conditions, and its main component is a fast and powerful moving platform that provides haptic feedback to the user [23].

### 2.4. Protocol

In order to prevent excessive movement artifact occurrences, the subjects were instructed to restrict sudden movements, talking, and laughing. Volunteers reported that they did not eat for 6 hours or drink for 2 hours before recording, so EGG was recorded in the fasting state. Additionally, the subjects confirmed that they had not used any medications in the past 6 months.

Prior to the placement of recording electrodes, adequate skin preparation was performed, i.e. shaving (if needed) with alcohol application or skin cleaning with abrasive gel (Nuprep, Weaver and Company, Aurora, CO, USA). Standard surface self-adhesive Ag/AgCl electrodes H135SG (Kendall/Covidien, Dublin, Ireland) were applied. For electrode placement, positions recommended in [21] were used:

- reference electrode—tissue covering the *iliac crest*;
- common electrode—on the stomach, 8 cm straight above the navel;
- channel 1 electrode—8 cm left of the common electrode inclined by 20 degrees from the line that connects the navel and the *sternum*;
- channel 2 electrode—8 cm left of the common electrode inclined by 55 degrees from the line that connects the navel and the *sternum*;
- channel 3 electrode—8 cm left of the common electrode inclined by 90 degrees from the line that connects the navel and the *sternum*.

Electrodes were placed above the dominant pacemaker region of the stomach rich with intestinal cells of Cajal. More specifically, channel 1 was placed towards the part of the stomach called the lesser curvature, while channel 3 was placed above the greater curvature region. The channel 2 electrode was placed between channels 1 and 3 [20].

EGG signals were continuously recorded during the session for approximately 30 minutes. The protocol was divided into the following four segments (approximate duration of each segment is provided in brackets):

- Test drive—in order to enable participants to become familiar with the driving simulator operation (~ 5 min);
- Resting sequence—baseline slow-wave activity before driving simulation was recorded (~ 5 min);
- Drive with motion—driving simulation with haptic feedback included (~ 5 min);
- Drive without motion—driving simulation with no haptic feedback (~ 5 min).

**Figure 2:**
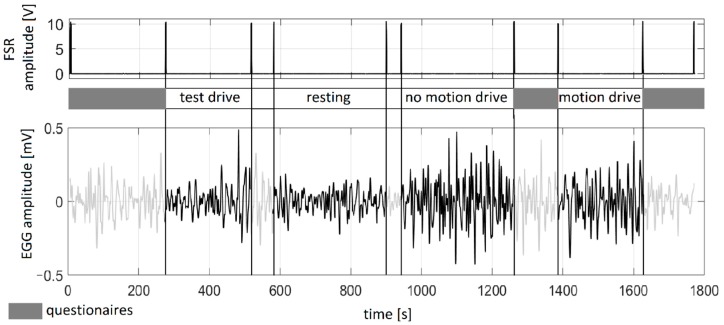
The top panel shows FSR that was used as a pushbutton for manual synchronization between driving sequences and EGG segments. The bottom panel presents a sample EGG signal in one subject. Between channels there is a timeline with annotations that show the order and duration of segments. Marked segments that correspond to the questionnaires were used for the SSQ (the first one prior to the recording, and the second two after the motion and no-motion drives), and for demographic data acquisition.

In order to counterbalance the testing protocol, the order of the last two sequences was altered for each volunteer. Between consecutive segments, the subjects were asked to complete a standard SSQ [7]. The participants were asked to rate 16 symptoms using a 4-point scale, where 0 = “none”, 1 = “slight”, 2 = “moderate” and 3 = “severe”. The total score ranging from 0 to 235.62 was calculated as a sum of weights for three groups of symptoms: nausea, oculomotor, and disorientation. The nausea sub-score ranging from 0.00 to 200.34 was also calculated based on the sum of weights for the nausea group of symptoms. Higher scores correspond to higher SS. The subjects completed the SSQ three times: prior to the recording and after both drives (with and without motion). Since the study was completed in Slovenia, the participants filled out a translated SSQ version in the Slovene language. The timeline of the recording protocol with a sample EGG signal and an illustration of FSR synchronization are presented in Figure 2.

### 2.5. EGG Analysis

A complete software analysis was performed in Matlab ver. R2013a (Mathworks Inc., Natick, MA, USA). In order to cancel out the frequency content outside the EGG range, EGG signals were filtered using Butterworth 3^rd^ order band-pass filter with cut-off frequencies from 0.03 to 0.25 Hz as suggested in [20].

#### 2.5.1. Motion Artifact Cancellation 

Recordings were obtained using three channels in order to increase robustness of the system and decrease sensitivity to motion artifacts. The first two steps in the analysis were:

- automatic selection of a channel that was least affected by artifacts, and
- manual cancellation of the remaining artifacts on the chosen channel.

For automatic channel selection, the amplitude range and power of the signal were calculated. A higher amplitude range in one of the channels suggested the presence of non-physiological peaks, and therefore signals from the channels that had the amplitude range 100% higher compared to the other channels were eliminated. In order to select a channel from the non-eliminated group of signals, the lowest power criterion was applied (the channel with the lowest power was selected for further analysis).

**Figure 3:**
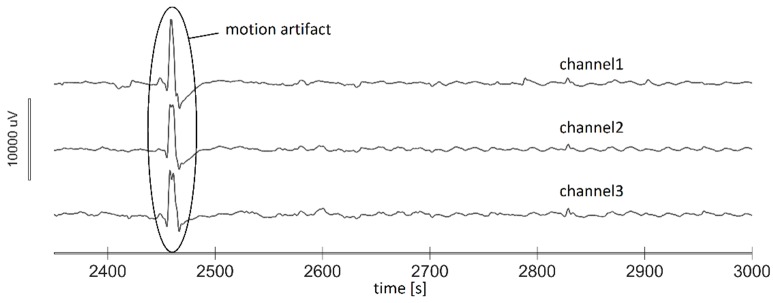
EGG signal with a large spike as a result of a movement artifact (in circle).

Although automatic channel selection is more desirable, it is stated in [24] that the most suitable way to detect and extract movement artifacts from EGG recording is educated observation. For example, sudden moves can induce relatively large spikes on all channels simultaneously (Figure 3.) unlike the physiological variations that display time lag between channels. Having these facts in mind, an experienced researcher examined all recorded EGG time series, visually detected all movement artifacts manually, and placed the corresponding markers. Following these markers, samples originating from movement artifacts were deleted, and the corresponding time portion was excluded from the analysis. 

#### 2.5.2. Feature Extraction

**Figure 4:**
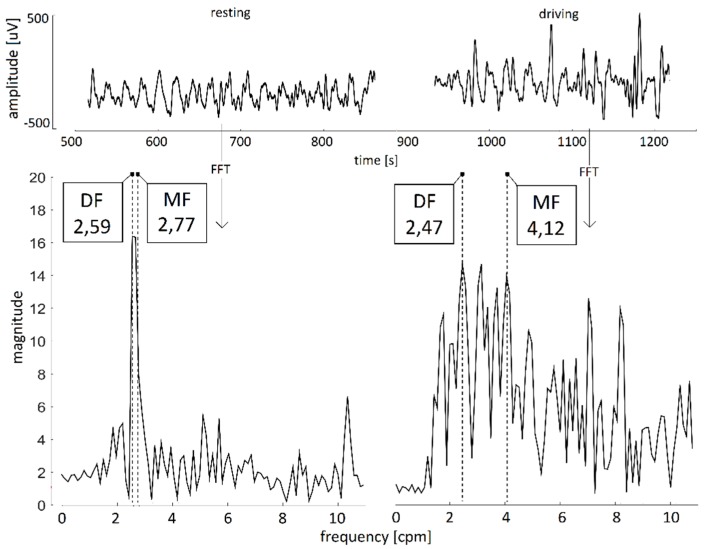
Illustration of difference between DF and MF parameters calculated from FFT of the EGG signal during resting and no-motion driving sequences.

DF was calculated as the position of the maximum peak in the frequency spectrum of the EGG signal [25]. MF was calculated as the frequency that divides the spectrum obtained by the application of Fast Fourier Transform (FFT) into two parts with the same power integral [18]. Both DF and MF were determined for each segment in the EGG time series. Figure 4. presents the DF and MF parameters of two sample signals from one test subject. We can visually observe changes in the frequency content during the driving sequence compared to the resting period. This change is the consequence of frequency shifts in FFT towards the tachygastric range (4–10 cpm). DF fails to detect this obvious shift because it is dependent on the global maximum peak in FFT. On the other side, MF reliably detects these frequency alterations and can be used as a quantitative measure of a frequency shift as suggested previously in [18]. Although the initial results on DF and MF sensitivity (Figure 4.) suggested that DF might be excluded, both parameters were kept in order to perform a detailed analysis on all recorded segments for final decision.

CF was obtained by dividing the magnitude of the maximum peak in the frequency spectrum by its RMS value. It was calculated for each segment in the EGG dataset with the aim to assess peak prominence in FFT. The percentage of spectral power in the normogastric range (2–4 cpm) of the EGG signal was derived from the power spectrum density (PSD) function calculated using Welch windowing. Additionally, for each segment in the EGG dataset, the RMS value was calculated. To determine a statistically significant difference between features calculated for various sequences, a paired-sampled t-test was applied. Results that had p-value < 0.05 were considered significant.

**Figure 5:**
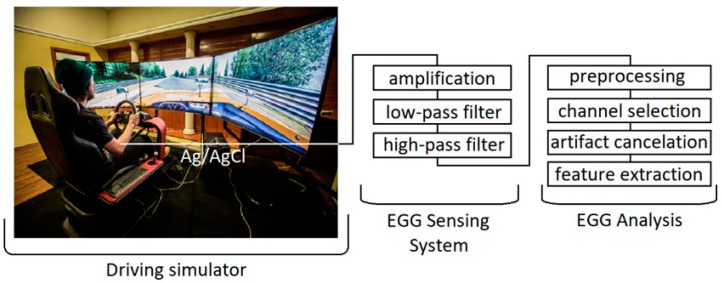
Nervtech’s 4DOF motion car driving simulator during driving simulation on the left panel. The EGG sensing system and steps performed for SS assessment (EGG analysis) are presented on the right panel.

In Figure 5 we sum up and present the complete system architecture comprising hardware and software components for SS assessment by the EGG sensing device in the Nervtech driving simulator.

## 3. Results

Four out of the 13 subjects (31%, IDN1-IDN4) were excluded from the study as their EGG signals were not suitable for the analysis. This was a consequence of the following problems:

- Drop-out due to severe nausea and anxiety symptoms (in one subject);
- Severe artifacts present in the signal, most probably due to the electrodes detachment and movements (in three subjects).

Feature extraction and further analysis was performed on EGG signals recorded in the remaining nine participants (ID1-ID9). Channel 1 (upper part of the stomach) was indicated as noise-free in one subject, and channels 2 and 3 (lower part of the stomach) were indicated as suitable for processing in eight subjects (4 for each channel).

**Figure 6:**
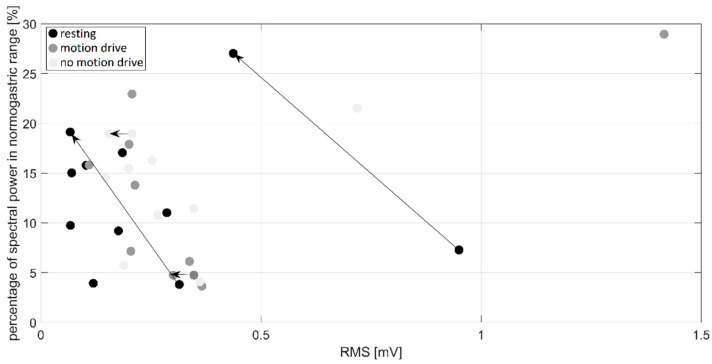
Scatter plot of RMS values and the percentage of spectral power in the normogastric range. Arrows connect points that are associated with the same EGG signal segments, before and after artifact cancellation.

Scatter plots of RMS values and the percentage of spectral power in the normogastric range for all segments (resting, motion drive, and no-motion drive) in nine subjects are presented in Figure 6. Segments that underwent manual artifact cancelation are presented with original (noisy) and corrected (noise-free) values connected with arrows in Figure 6. Artifact cancellation was performed for four different and independent segments: (1) in ID2 and ID5 for resting; (2) in ID3 for motion drive; and (3) in ID4 no-motion drive.

**Figure 7:**
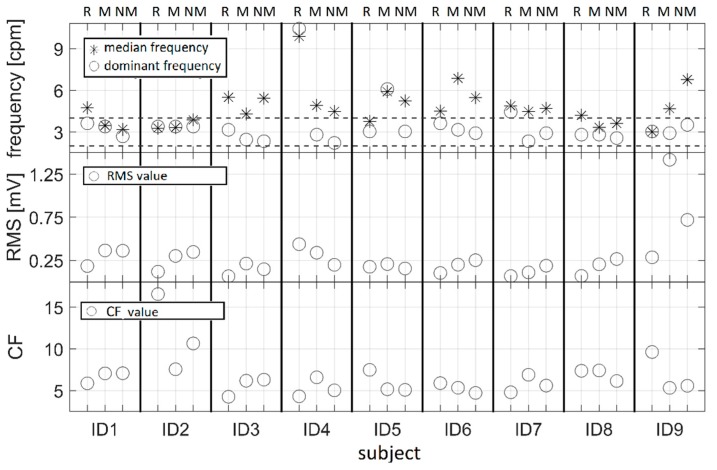
Dominant frequencies, median frequencies (upper panel), RMS (middle panel), and CF (bottom panel) values calculated for all sequences during EGG recordings and obtained in 9 subjects (ID1-ID9). Abbreviations R, M, and NM stand for resting, motion, and no motion.

**Figure 8:**
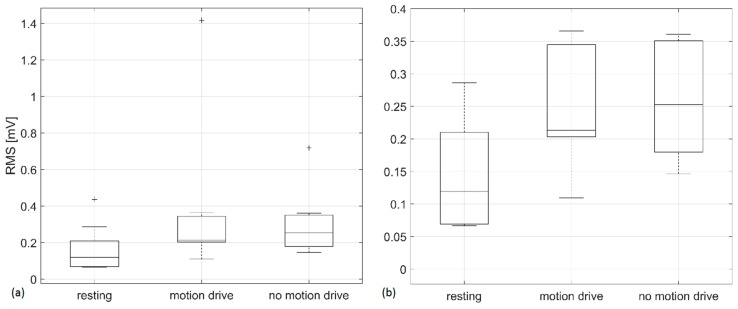
Box plots presented for RMS values divided into three groups: resting, motion, and no motion (**a**). A zoomed box plot is presented on (**b**) (from 0 to 0.4 mV).

Graphical representation of DF and MF values is shown in Figure 7 (upper panel), while on the middle and bottom panels RMS and CF values, are shown for nine subjects respectively (ID1-ID9). Box plots of RMS values for three segments (resting, motion drive, and no-motion drive) are presented in Figure 8.

A statistically significant difference was obtained only between RMS values for resting and no-motion drive sequences (p = 0.03). For the other calculated features (DF, MF, CF and the percentage of spectral power in the normogastric range), a paired-sampled t-test did not give any significant results regarding variation between different sequences. Although the results presented in Figure 8 suggest a distinction between resting and the motion drive, there was no statistically significant difference between resting and the motion drive (p = 0.10). These results should be taken with special precaution, since data from only nine subjects was used for statistical tests.

The results of the SSQ for the nine subjects that successfully finished the test protocol (ID1-ID9) are presented in Table 3 together with the SSQ results for the remaining four subjects (IDN1-IDN4). The total SSQ scores indicate that higher levels of SS were experienced only by ID9 after the no-motion drive, whereas higher levels of SS were experienced by ID4, ID5, and ID9 after the motion drive. Higher total SSQ scores were followed by higher nausea sub-scores. In case that a participant rates all symptoms with none, mild, moderate, or severe, total SSQ scores are 0, 78.54, 157.08, and 235.62, respectively. In case of nausea sub-scores, the values that correspond to all none, mild, moderate, or severe symptoms are 0, 66.78, 133.56, and 200.34, respectively. This implies that the majority of our subjects (see Table 3) had none to mild symptoms.

## 4. Discussion

In this section learned lessons are discussed by following the order of appearance of topics as presented in Section 3: Methods.

### 4.1. Lessons Learned: Open-Source EGG 

This study demonstrated that our open-source EGG sensing system can be used for the acquisition of EGG activity signals during driving simulation. There are a few issues that need to be addressed for the future device improvement. Firstly, the device should be realized on a printed-circuit board (PCB) to make it more resistant to external noises and also to make it more suitable for transportation and less vulnerable to hazards that may occur during manipulation. The implementation of a HP filter with an extremely low cut-off frequency as presented here (0.016 Hz) required high-resistance values (10 MΩ) that may produce current leakage in PCB implementation, which is why alternative filtering designs should be considered.

### 4.2. Lessons Learned: Protocol

The percentage of recording sessions that were not successfully obtained and did not provide EGG signals suitable for further analysis is 31%, which is not insignificant. We identified and discussed reasons that caused unreliable EGG recordings:

- Firstly, subject’s movements can cause erroneous EGG, so body movements should be carefully controlled when recording EGG in a dynamic environment, such as driving simulation with haptic feedback.
- Secondly, EGG could be affected by the posture as stated in [26]. Although the supine position is more preferable than the sitting position, the results presented in [18] showed that successful EGG assessment can be performed in the sitting position. We assumed that the posture did not significantly affect our recordings.
- Thirdly, we faced a problem with electrode detachment in two study participants, which caused the drop-out of those two subjects. Hence, we propose the application of an additional protective layer of adhesive bandage over the surface electrodes.

In this study, the resting sequence was recorded after the test drive (see Figure 2), which was a mandatory part of the protocol for the subjects to get acquainted with the simulator. However, that could affect the regular slow wave expected during the resting sequence. For future work, we recommend the resting sequence prior to all the other, or a longer pause after the test drive in order to have reliable baseline recording. The test drive probably influenced CF. Higher CF values indicate an EGG spectrum with a prominent peak. This peak-prominent spectrum shape corresponds to the resting sequence with a dominant normogastric rhythm [18]. It is expected that the driving sequence should have lower CF values and less prominent peak(s) as shown in Figure 4. The results presented in Figure 7 are not in accordance with this expectation. 

### 4.3. Lessons Learned: Channel Selection

Channel 2 was previously recommended in [21] as the most suitable for EGG recording in the supine position. However, the current research showed no significant difference in the acquired signals from channels 2 and 3. Further, in this study, the signal from channel 1 proved to be useful for only one subject (11%), which implies that it can be excluded in similar studies. This is in contrast with results presented in [18] where the signal from channel 1 was used for further processing in all subjects. These opposite findings could be the consequence of different dynamics since the study presented in [18] was recorded during static conditions. The most important lesson learned from this testing procedure is that there is a benefit from the application of more than one channel for dynamic EGG recording.

### 4.4. Lessons Learned: Artifact Cancellation

Out of the 27 signal sequences analyzed (9 subjects × 3 sequences), motion artifacts were visually detected in four of them (16%). Following the recommendations of [24], our experienced researcher deleted marked samples. In all segments that were additionally processed, we observed a decrease in RMS (Figure 6). Therefore, these artifacts had a higher amplitude than slow waves, and if not excluded they could lead to false conclusions regarding signal power. For two sequences we can see a drastic increase in the percentage of spectral power in the normogastric range, particularly for resting sequences. This implies that motion artifacts can completely alter the frequency content, especially when artifact-free slow wave during baseline recording is expected.

### 4.5. Lessons Learned: Feature Extraction

While DF is a commonly used EGG parameter [12,21], its indication of dysrhythmia is questionable. Based on previous visual inspection of DF (Figure 4), it can be concluded that DF does not provide reliable information about FFT spectrum changes. On the contrary, MF provides a much more meaningful estimation of the spectrum variation (Figure 4). When calculated on all recorded EGG sequences, it was concluded that DF was within the normogastric range for 17 out of 18 (95%) driving sequences, while MF was in that range for only six out of 18 (33%). This indicates higher variability, i.e. sensitivity of MF, across driving sequences. It should be mentioned that in [18] MF failed to indicate any alteration between resting and virtual reality sequences that induced nausea. These contradictory results suggest that further investigation of the MF feature should be considered. To sum up, based on these results, DF may be more suitable for EGG with a normal spectrum (resting sequence), while MF could be an option for dispersed frequency content.

CF can be used to assess the prominence of peaks [27], so it could be useful to discriminate EGG recordings with a normal slow wave (clear dominant peak in the spectrum) from unusual ones (sparse spectrum). Previous results suggested that CF decreases as a result of nausea [18]. In this paper, we did not find any consistent drop in CF for driving sequences compared to a resting state. This could be a consequence of non-ideal conditions for the resting protocol already discussed in 4.2., which results in the absence of a dominant peak with drastically higher values than the rest of the spectrum.

We used the RMS value as an estimator of power changes in EGG signals since it has been proved that RMS increase correlates with nausea occurrence [18,28]. The results presented in Figure 7 support these findings since RMS increased for seven out of nine (78%) subjects during the driving sequence. Although ID4 scored high on the SSQ (Table 3), RMS failed to detect nausea (Figure 7). This could be the consequence of an inappropriately recorded resting sequence (see 4.2 and DF in Figure 7). As expected, ID5 and ID9 showed both higher SSQs and RMSs during driving sequences (see Table 3 and Figure 7). Overall, box plots presented in Figure 8 show that there is a general tendency towards RMS increase during the application of a driving simulator compared to the RMS values obtained during the resting sequences. This parameter should be used in the future for EGG-based SS assessment, and its normalization should be considered as suggested in [18].

The percentage of spectral power in the normogastric range presented in Figure 6 did not show any differences between recording sequences. This could be a consequence of short recording sequences and the fact that the majority of our subjects did not report any nausea symptoms.

The absence of statistically significant results may be a consequence of a relatively small study group. That is why we presented a box plot only for RMS values–the parameter that showed a statistical significance between resting and the no-motion drive.

### 4.6. Lessons Learned: Haptic Feedback in Relation to SS

There was no noticeable difference in the observed parameters between the no-motion and motion driving session in all parameters. Although it was previously concluded that haptic feedback could reduce SS [20], our protocol did not induce any severe symptoms of nausea in nine of the subjects that were analyzed, which is why these results are expected.

We did not encounter any significant artifacts in EGG signals related to haptic feedback. Therefore, our EGG open-source system does not have contraindications for this kind of application.

### 4.7. Lessons Learned: Qualitative and Quantitative Nausea Assessment

The SSQ revealed that the proposed protocol induced moderate sickness symptoms in three study participants (Table 3):

- ID4 after driving with motion,
- ID5 after driving with motion, and
- ID9 after both driving with and without motion.

ID5 revealed a slight RMS increase during the driving sequence, while ID9 had a higher RMS increase for both driving sessions compared to the resting period. These RMS values in ID9 can be observed in Figure 6 and Figure 7 as extreme values on the abscissa. Additional visual observation of the time series recorded in subject ID9 did not reveal any artifacts that could affect RMS. Therefore, higher values were a consequence of a drastic amplitude increase that could be correlated with nausea symptoms reported by SSQ total scores (Table 3).

A decrease in RMS in subject ID4 for driving sequences was unexpected. This could be a consequence of signal irregularity during the resting sequence–RMS, MF and DF were higher than expected (see 4.5).

It should be mentioned that subject IDN3 reported severe nausea during the motion drive. Therefore, the recording was not completed and the SSQ was not filled out. These signals were not included in the statistical analysis due to the presence of excessive motion artifacts being a result of subject’s exit from the driving simulator.

In order to provide more reliable recommendations on the relationship between SS symptoms and EGG features, it is necessary to have a larger study group and a protocol that has a higher probability of nausea induction.

## 5. Conclusions

Based on the presented results and previous discussion, the following answers to the research questions are proposed:

- Recording of EGG during driving simulation is possible using our custom-made open-source device with careful considerations regarding recording setup and the protocol. Despite that, its effectiveness for SS assessment is yet to be shown.
- RMS values might be used for the estimation of amplitude variations in EGG signals. Correlation between RMS and nausea should be examined in a future study. MF, CF, and DF could be used for the assessment of the EGG spectrum. In order to confirm these findings, future study should be performed on a larger sample.
- There was no clear difference between resting, motion, and no-motion sequences, except for an increase in RMS for driving sessions compared to resting.

Recommendations for future research are:

- EGG under dynamic conditions should be recorded by carefully following protocol recommendations and the application of more than one EGG channel.
- The resting sequence of EGG recording should be obtained prior to any simulator activity.
- EGG signals should be visually examined in order to detect and manually extract motion artifacts.
- Features for a description of frequency content (DF, MF, and CF) should be carefully examined prior to any conclusions.
- Further improvement of the EGG device, primarily realization on a PCB with consideration regarding filter design.
- Assessment of nausea on a larger study group with higher statistical power in order to divide subjects into nausea and non-nausea groups. For the selection of target population, it could be beneficial to use a Motion Sickness Susceptibility Questionnaire to estimate susceptibility to suffer from SS.

## Figures and Tables

**Table 1:** Demographic data for subjects included in the study, with information about their driving and simulation experience. Only participants with ID1-ID9 were included in the EGG analysis. Participants with IDN1-IDN4 were excluded from the study (see Section 3 for more details).

Subject	Age [years]	Sex [F-Female, M-Male]	Height [cm]	Weight [kg]	Driving Experience [years]	Driving Simulator Experience [Yes/No]
ID1	23	F	173	60	5	Yes
ID2	23	M	172	60	5	No
ID3	26	F	169	56	8	No
ID4	23	M	180	88	4	No
ID5	32	M	192	115	14	Yes
ID6	47	M	182	87	29	No
ID7	23	M	173	65	5	Yes
ID8	40	F	160	49	15	Yes
ID9	25	F	169	59	6	Yes
IDN1	26	M	183	97	6	Yes
IDN2	27	M	181	75	9	Yes
IDN3	33	M	177	60	15	Yes
IDN4	35	M	186	78	17	No

**Table 2:** Components for the EGG open-source device.

Amplification		LP Filtering			HP Filtering		
Instrumentation amplifier	R_g_	Operational amplifier	R_lp_	C_lp_	Operational amplifier	R_hp_	C_hp_
INA114BP	50 Ω	TL072CP	15 kΩ	2.2 µF	TL072CP	10 MΩ	1 µF

**Table 3:** Simulator Sickness Questionnaire (SSQ) results for 13 subjects. ID1-ID9 are subjects included in the EGG analysis. IDN1-IDN4 are subjects excluded from the study (see the main text for more details). Shaded subjects experienced higher SS symptoms, as revealed by the SSQ.

Subject	Resting		No-Motion Drive		Motion Drive	
	Nausea	Total	Nausea	Total	Nausea	Total
ID1	28.6	49.2	0.0	19.0	0.0	34.2
ID2	28.6	30.0	28.6	18.8	/	/
ID3	9.5	15.0	9.5	31.5	19.1	22.7
ID4	19.1	11.3	28.6	37.6	38.2	68.0
ID5	38.2	18.7	38.2	15.1	57.2	68.1
ID6	0.0	7.6	19.1	15.0	19.1	7.5
ID7	28.6	22.6	28.6	7.5	9.5	7.5
ID8	0.0	11.2	9.5	11	0.0	0.0
ID9	9.5	26.4	76.3	117.4	47.7	71.8
IDN1	47.7	68.1	57.2	83.2	57.2	56.5
IDN2	0.0	0.0	19.0	18.9	0.0	0.0
IDN3	9.5	7.5	38.2	49.2	/	/
IDN4	0.0	0.0	0.0	0.0	9.5	3.7
